# A Manual of the Practice of Medicine

**Published:** 1900-12

**Authors:** 


### Books and Magazines.

---

A Manual of the Practice of Medicine.—Prepared especially for students by A. A. Stevens, A. M., M. D., Professor of Pathology in the Women's Medical College of Pennsylvania; Lecturer on Terminology and Instructor in Physical Diagnosis in the University of Pennsylvania, etc. Fifth edition; revised and enlarged. Illustrated. 519 pages. Price, flexible leather, $2.00. Philadelphia: W. B. Saunders, publisher, 925 Walnut Street.

This book is one of the best manuals we have. The arrangement of the subject matter is all that could be demanded. The present edition has been thoroughly revised, and much new matter added. More elaborate discussion has been given to the diseases of the pancreas, the blood and the ductless glands. The articles on appendicitis, angina pectoris, aphasia, myxedema and syringo-myelia have been entirely rewritten. The book altogether is to be recommended for quick reference, and especially for students. T. J. B.

